# Testosterone Enhances K_V_ Currents and Airway Smooth Muscle Relaxation Induced by ATP and UTP through P2Y_4_ Receptors and Adenylyl Cyclase Pathway

**DOI:** 10.3390/ijms25094652

**Published:** 2024-04-24

**Authors:** Abril Carbajal-García, Jorge Reyes-García, Verónica Díaz-Hernández, María F. Casas-Hernández, Francisco Javier Flores-Murrieta, Luis M. Montaño

**Affiliations:** 1 Departamento de Farmacología, Facultad de Medicina, Universidad Nacional Autónoma de México, Mexico City 04510, Mexico; carbajalabril@gmail.com (A.C.-G.); reyes.garcia.jorge@gmail.com (J.R.-G.); fercasashdz@hotmail.com (M.F.C.-H.); 2 Departamento de Embriología, Facultad de Medicina, Universidad Nacional Autónoma de México, Mexico City 04510, Mexico; roveazdih@yahoo.com.mx; 3 Unidad de Investigación en Farmacología, Instituto Nacional de Enfermedades Respiratorias Ismael Cosío Villegas, Mexico City 14080, Mexico; fjfloresmurrieta@yahoo.com.mx; 4 Sección de Estudios de Posgrado e Investigación, Escuela Superior de Medicina, Instituto Politécnico Nacional, Mexico City 11340, Mexico

**Keywords:** testosterone, airway smooth muscle, ATP, UTP, K^+^ currents, smooth muscle relaxation, asthma

## Abstract

Numerous studies suggest the involvement of adenosine-5′-triphosphate (ATP) and similar nucleotides in the pathophysiology of asthma. Androgens, such as testosterone (TES), are proposed to alleviate asthma symptoms in young men. ATP and uridine-5′-triphosphate (UTP) relax the airway smooth muscle (ASM) via purinergic P2Y_2_ and P2Y_4_ receptors and K^+^ channel opening. We previously demonstrated that TES increased the expression of voltage-dependent K^+^ (K_V_) channels in ASM. This study investigates how TES may potentiate ASM relaxation induced by ATP and UTP. Tracheal tissues treated with or without TES (control group) from young male guinea pigs were used. In organ baths, tracheas exposed to TES (40 nM for 48 h) showed enhanced ATP- and UTP-evoked relaxation. Tetraethylammonium, a K^+^ channel blocker, annulled this effect. Patch-clamp experiments in tracheal myocytes showed that TES also increased ATP- and UTP-induced K^+^ currents, and this effect was abolished with flutamide (an androgen receptor antagonist). K_V_ channels were involved in this phenomenon, which was demonstrated by inhibition with 4-aminopyridine. RB2 (an antagonist of almost all P2Y receptors except for P2Y_2_), as well as N-ethylmaleimide and SQ 22,536 (inhibitors of G proteins and adenylyl cyclase, respectively), attenuated the enhancement of the K^+^ currents induced by TES. Immunofluorescence and immunohistochemistry studies revealed that TES did not modify the expression of P2Y_4_ receptors or COX-1 and COX-2, while we have demonstrated that this androgen augmented the expression of K_V_1.2 and K_V_1.5 channels in ASM. Thus, TES leads to the upregulation of P2Y_4_ signaling and K_V_ channels in guinea pig ASM, enhancing ATP and UTP relaxation responses, which likely limits the severity of bronchospasm in young males.

## 1. Introduction

Extracellular adenosine 5′-triphosphate (ATP) and other related nucleotides are involved in the pathogenesis of respiratory diseases such as chronic obstructive pulmonary disease (COPD) and asthma through abnormal purinergic signaling [1,2,3,4,5,6]. Patients with COPD have increased ATP levels in bronchoalveolar lavage fluid (BALF), which are linked to poor lung function, and show an increase in neutrophilic inflammation due to the upregulation of ATP-activated purinergic receptors [7,8]. Moreover, induction of the purinergic pathway can stimulate human neutrophils to produce interleukin (IL)-8, a key mediator associated with asthma and COPD [9,10,11]. In the case of asthma, ATP plays a multifaceted role in causing airway inflammation, bronchoconstriction, mucus secretion, and airway remodeling [12,13,14,15]. Increased ATP levels have been detected in the airways of asthmatics and mouse models of experimentally induced asthma after allergen exposure [1,13,16]. Elevated ATP and uridine 5′-triphosphate (UTP) exert chemotactic effects on airway dendritic cells that induce allergic sensitization [13,17]. Several cell types, including epithelial cells, inflammatory cells, and neuronal terminals, release ATP when inflammatory and hypoxic conditions prevail, which is the case in most severe acute asthma attacks [18,19,20,21,22]. ATP sustains and enhances airway inflammation in asthma via activating dendritic cells and producing Th2 cytokines [13,16]. This nucleotide can also induce airway remodeling in asthmatic mice via the upregulation of P2X4 receptors [23]. In addition, purinergic signaling molecules, such as adenosine, ATP, and uridine 5′-triphosphate (UTP), are key mediators in the modulation of airway smooth muscle (ASM) tone [14,24,25,26,27,28].

Purinergic signaling involves the activation of purinergic receptors. These proteins are classified into two major subtypes: P1 (adenosine) and P2 receptors. P2 receptors are divided into P2X ionotropic receptors (P2X1-7) and P2Y metabotropic receptors (P2Y_1,2,4,6,11–14_) [29]. P2X receptors are only activated by ATP, leading to rapid cation movement across the cell membrane. On the other hand, activation of P2Y receptors by ATP, UTP, ADP, or UDP triggers intracellular signaling cascades via G protein coupling [30]. Although ATP is associated with airway hyperresponsiveness (AHR) and bronchospasm, some studies have shown that this nucleotide triggers a biphasic response in the tone of the ASM [27,31]. Initially, ATP induces the ASM contraction via intracellular Ca^2+^ increments [14,24]. This purinergic mediator is also released alongside acetylcholine as a co-transmitter from the parasympathetic nerves [32,33]. It causes mast cells to degranulate [34] and results in the release of histamine [35] and leukotrienes [28], thereby enhancing bronchoconstriction. However, once the maximal contraction is reached, relaxation of the tissue takes place [27]. The evidence postulates that the synthesis of prostaglandin E_2_ (PGE_2_) and the desensitization of purinergic receptors are responsible for the relaxation phase [27,36,37]. Using guinea pig tracheal tissue, we have characterized the role of P2Y_2_ and P2Y_4_ receptors in mediating smooth muscle relaxation after stimulation with ATP or UTP [27]. The activation of these receptors triggers signaling cascades leading to the opening of K^+^ channels, particularly voltage-dependent delayed K^+^ (K_V_) channels and high-conductance Ca^2+^-activated K^+^ (BK_Ca_) channels, facilitating membrane hyperpolarization and subsequent ASM relaxation [27]. This complex process, and particularly the opening of K^+^ channels, is modulated by adenylyl cyclase (AC), cyclic adenosine monophosphate (cAMP), and the protein kinase A (PKA) signaling pathway (this enzyme phosphorylates K^+^ channels and triggers their opening) [38,39,40,41], highlighting the intricate regulatory mechanisms involved in ATP-induced ASM relaxation [27,37].

Androgens, particularly testosterone (TES), have garnered increased attention for their potential protective factor against asthma. This sex hormone reduces airway inflammation, promotes larger airway caliber, decreases ASM hyperresponsiveness, and relieves asthma symptoms, especially in teenage boys and young men, when plasma TES levels rise from 6 to 50 nanomolar (nM) [42,43,44,45]. Androgens utilize both genomic and non-genomic mechanisms to elicit their physiological effects. In a temporal spectrum spanning hours to days, these hormones cross the plasma membrane and interact with the androgen receptor (AR) located in the cytosol, causing genomic responses characterized by transcriptional changes and protein synthesis [46]. In contrast, the non-genomic effects of TES manifest independently of its binding to the androgen receptor and occur rapidly, within a time frame of seconds to minutes [47,48]. Several studies have provided evidence that TES has direct non-genomic effects on ASM relaxation [48,49,50,51,52]. These effects are mainly related to the blockade of membrane Ca^2+^ channels, including L-type voltage-dependent Ca^2+^ channels (L-VDCCs) and store-operated Ca^2+^ channels (SOCCs) [49,51,52]. Blocking Ca^2+^ channels results in a reduction in the intracellular Ca^2+^ concentration ([Ca^2+^]_i_), consequently inducing the relaxation of the ASM [53,54,55].

In addition to Ca^2+^ channels, K^+^ channels play a primary role in regulating smooth muscle cells’ membrane potential and excitability, influencing airway tone and contractility [27,56,57]. Our recent studies demonstrated that TES can directly modulate the expression of K_V_1.5 and K_V_1.2 in guinea pig ASM and enhance the relaxation response to salbutamol and theophylline via a genomic pathway [40,58]. Moreover, this androgen augments the expression of the β_2_ adrenergic receptor (β_2_-AR) in the ASM [40]. Based on our previous studies, we aimed to investigate the influence of TES on ATP- and UTP-induced relaxation and the role of K^+^ channels in this response, using guinea pig tracheal rings as an experimental model. We also explored other possible molecular mechanisms underlying the TES-induced enhancement of ATP- and UTP-evoked ASM relaxation, focusing on purinergic signaling pathways and regulation of K_V_ channels. Our experimental results provide new insights into the interactions between TES, purinergic signaling pathways, and the activity of K^+^ channels in the ASM, which can lead to potential therapeutic targets for airway diseases such as asthma.

## 2. Results

### 2.1. Testosterone Increases the Relaxation Response to ATP and UTP in Guinea Pig Tracheal Rings

**Figure 1:**
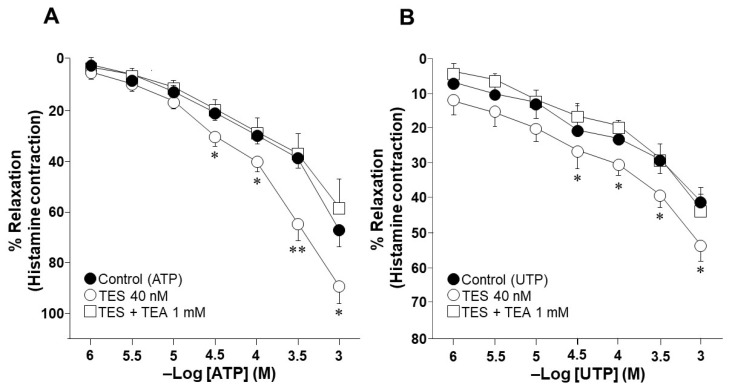
Effect of testosterone (TES) and tetraethylammonium (TEA) on ATP- and UTP-induced relaxation of guinea pig tracheal rings. (**A**) The guinea pig airway smooth muscle (ASM), previously constricted with histamine (His, 10 µM), was relaxed after the cumulative addition of ATP (1, 3.2, 10, 32, 100, 320, and 1000 µM). The concentration–response curve shifted to the left, indicating that a 48 h incubation with TES (40 nM) improved the relaxation response to ATP. Compared to the control group, tissues exposed to TES showed enhanced ASM relaxation at ATP 32, 100, 320, and 1000 µM. The effect of TES was abolished by the addition of TEA (1 mM), a nonspecific K^+^ channel blocker, suggesting that these channels are involved in the potentiation of smooth muscle relaxation. Symbols represent the mean values ± the standard error of the mean (S.E.M). ** *p* < 0.01, * *p* < 0.05. A one-way analysis of variance followed by a Dunnett’s test was performed (*n* = 9). (**B**) In a concentration-dependent manner, stimulation with cumulative concentrations of UTP (1, 3.2, 10, 32, 100, 320, and 1000 µM) relaxed tracheal tissues that had been pre-contracted with His (10 µM). Incubation with 40 nM TES for 48 h shifted the UTP relaxation curve to the left. TEA (1 mM) inhibited the increase in UTP-induced ASM relaxation, indicating the involvement of K^+^ channels in the effect of TES. * *p* < 0.05. A one-way analysis of variance was performed followed by a Dunnett’s test (*n* = 7).

The cumulative addition of ATP or UTP resulted in a concentration-dependent relaxation of guinea pig tracheal rings. ATP caused a stronger relaxation response, reaching up to 66.98 ± 8.6% of the histamine contraction response. In comparison, UTP only reached 40.9 ± 4% of relaxation with respect to the tissue contraction induced by 10 µM histamine (Figure 1). The exposure of tracheal rings to 40 nM TES for 48 h markedly enhanced the relaxation response induced by ATP and UTP. Significant differences were observed when 32, 100, 320, and 1000 µM ATP (Figure 1A) or UTP (Figure 1B) were added to the organ bath chambers. The maximum concentration of ATP used (1000 µM) in the presence of TES reached 89.15 ± 7.1% of relaxation, while UTP 1000 µM induced the relaxation of the tracheal tissues up to 53.21 ± 4.77% in the presence of the androgen. The nonspecific blocker of K^+^ channels, tetraethylammonium (TEA, 1 mM), completely prevented the effect of TES. These results suggest that the androgen action involves the regulation of K^+^ channels, as the TES-induced enhancement of relaxation is abolished in the presence of TEA.

### 2.2. Testosterone Increases ATP- and UTP-Induced K^+^ Currents in Airway Myocytes through a Genomic Pathway

**Figure 2:**
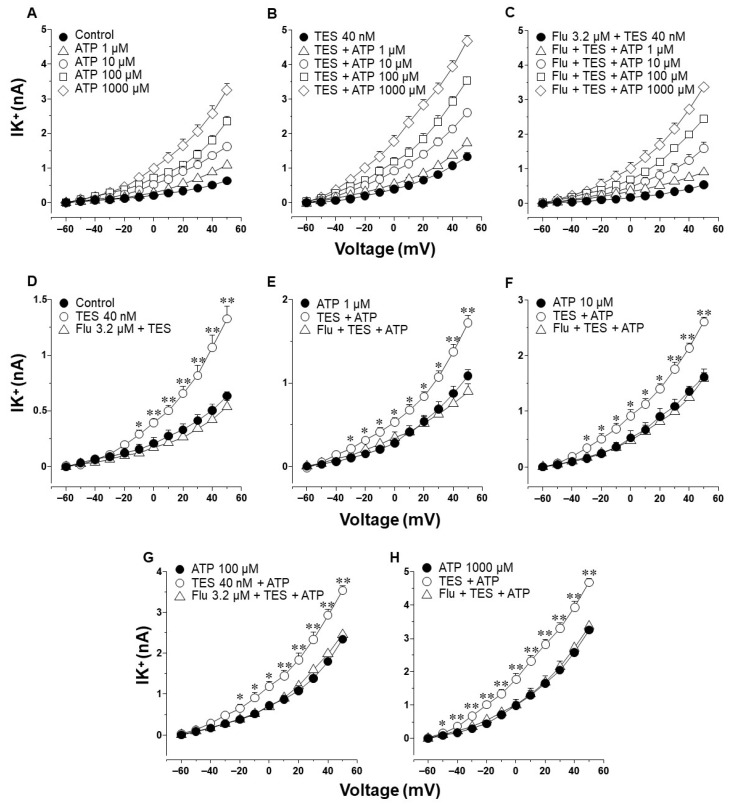
Testosterone (TES) enhances ATP-induced K^+^ currents through a genomic effect in guinea pig tracheal myocytes. Serial depolarization steps from −60 to +50 mV in increments of 10 mV were applied to single cells to elicit outward K^+^ currents (IK^+^). (**A**) Guinea pig tracheal myocytes perfused with increasing concentrations of ATP 1 µM, 10 µM, 100 µM, and 1000 µM showed an increase in the IK^+^ (*n* = 6). (**B**) The ATP-induced IK^+^ were higher in smooth muscle cells treated with 40 nM TES for 48 h than in the control group (*n* = 7). (**C**) Flutamide (Flu 3.2 µM, androgen receptor antagonist; *n* = 7) abolished this effect. Statistical analysis of the individual ATP concentrations tested in the three experimental groups (control cells without TES, cells exposed to TES, and cells incubated with Flu and TES) is shown in (**D**–**H**), respectively. The data indicate that the cells chronically exposed to TES 40 nM had a higher increase in the IK^+^ caused by ATP and depolarizing pulses. Flu annulated this effect. Different scales were used for the *Y*-axis in (**D**–**H**) to show the statistical differences. An analysis of variance followed by Dunnett’s tests was performed. Symbols represent the mean values ± S.E.M. In (**D**), * *p* < 0.05, ** *p* < 0.01 comparing TES group vs. control or Flu groups. In (**E**–**H**), * *p* < 0.05, ** *p* < 0.01 comparing TES + ATP group vs. ATP or Flu + TES + ATP group.

**Figure 3:**
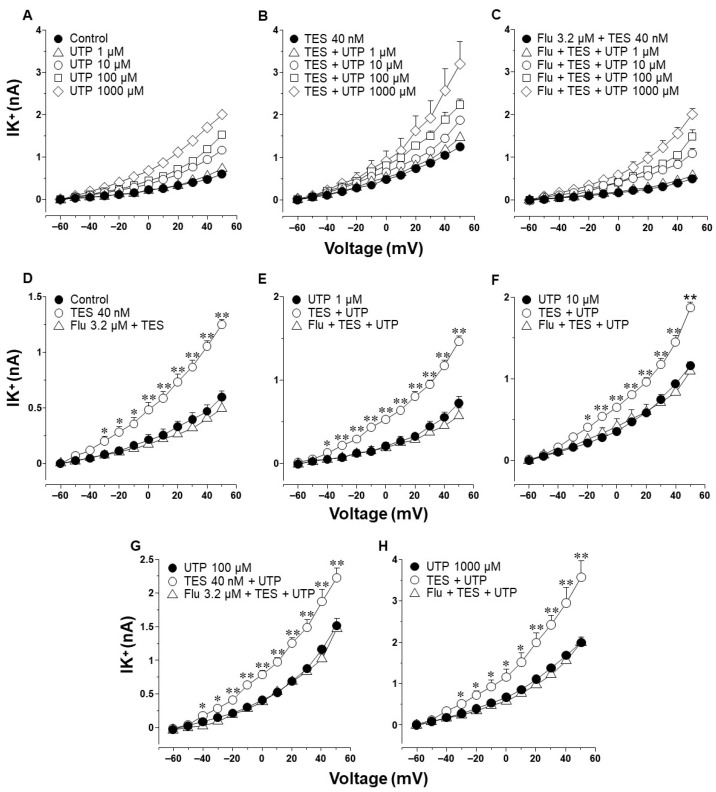
The agonist of P2Y receptors, UTP, generates K^+^ currents that are amplified by testosterone (TES) via the androgen receptor signaling. (**A**) Depolarizing pulses ranging from −60 mV to +50 mV were applied to guinea pig tracheal myocytes. These pulses generated outward K^+^ currents (IK^+^) which displayed an increase in response to UTP (1 µM, 10 µM, 100 µM, and 1000 µM) in a concentration-dependent manner; *n* = 6. (**B**) The treatment of smooth muscle cells with TES 40 nM for 48 h enhanced the UTP-induced IK^+^ (*n* = 6), while flutamide (Flu, 3.2 µM, the androgen receptor antagonist) abolished this effect, *n* = 6, (**C**). Figures (**D**–**H**) display the statistical analysis of the individual UTP concentrations used in the different experimental groups (control without TES, TES, and Flu + TES). Notice that Flu hindered the TES-induced potentiation of IK^+^ when they were triggered by depolarizing pulses and/or UTP. Figures (**D**–**H**) use different *Y*-axis scales to illustrate statistical significance. Note that the IK^+^ produced by each tested UTP concentration is smaller than the IK^+^ elicited by ATP. Symbols represent the mean values ± S.E.M. Analysis of variance followed by Dunnett’s test was used for statistical analysis. In (**D**), * *p* < 0.05, ** *p* < 0.01 comparing TES group vs. control or Flu groups; in (**E**–**H**), * *p* < 0.05, ** *p* < 0.01 comparing TES + UTP vs. UTP or Flu + TES + UTP groups.

Since K^+^ channels are implicated in the ATP and UTP relaxation responses [27], we conducted patch-clamp experiments to investigate whether TES could modify the K^+^ currents induced by these nucleotides. In these experiments, single myocytes were subjected to a depolarizing-pulse protocol from −60 to +50 mV. This protocol generated outward K^+^ currents that, under control conditions, peaked at ~0.6 nanoamperes (nA) at the maximum voltage tested (Figure 2A and Figure 3A, closed circle). The addition of increasing concentrations of ATP or UTP (1, 10, 100, and 1000 µM) over the depolarization step protocol augmented the K^+^ currents in a concentration-dependent manner (Figure 2A and Figure 3A). The highest ATP concentration tested produced a K^+^ current of approximately 3.3 nA (Figure 2A,H), while UTP 1000 µM reached a peak of ~2 nA (Figure 3A,H). Exposure of tracheal myocytes to TES 40 nM markedly increased the K^+^ currents generated by perfusion of ATP or UTP (Figure 2B and Figure 3B). The androgen receptor antagonist flutamide (Flu) reversed the enhancement of ATP- and UTP-induced K^+^ currents (Figure 2C and Figure 3C). To analyze the effects of TES and Flu appropriately, the increase in the K^+^ currents was compared concerning the tested concentration of ATP or UTP. Figure 2D and Figure 3D illustrate that TES markedly augmented the K^+^ currents induced by the protocol of depolarizing pulses alone from −10 mV and −30 mV ahead, respectively, and flutamide annulled this effect. These findings indicate a direct regulation of the androgen over K^+^ channels. Adding ATP or UTP at several concentrations (1, 10, 100, and 1000 µM) increased the K^+^ currents over the depolarizing steps protocol (Figure 2E–H and Figure 3E–H). Moreover, TES significantly improved the ATP- and UTP-induced K^+^ currents from −50 mV (Figure 2H), −40 mV (Figure 3E,G), −30 mV (Figure 2E,F and Figure 3H), or −20 mV ahead (Figure 2G and Figure 3F). Flutamide reversed this effect. Overall, these results confirm the genomic nature of the influence of TES on ATP- and UTP-induced K^+^ currents and the importance of P2Y receptors for generating K^+^ currents.

### 2.3. Voltage-Dependent Delayed Rectifier K^+^ (K_V_) Channels Are Responsible for Testosterone Enhancement of ATP- and UTP-Induced K^+^ Currents

**Figure 4:**
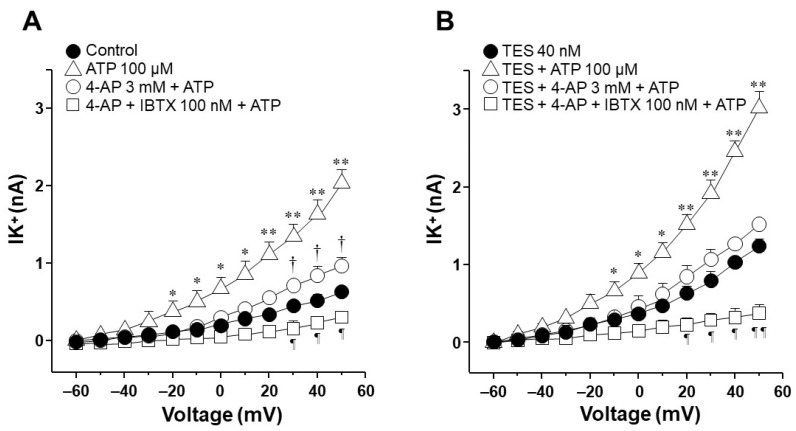
Voltage-dependent delayed rectifier K^+^ (K_V_) channels are involved in the potentiating effect of testosterone (TES) on ATP-induced K^+^ currents. (**A**) Depolarizing pulses from −60 to +50 mV in 10 mV increments produced an outward K^+^currents (IK^+^) that peaked at approximately 0.7 nanoamperes (nA) (control, closed circle). The addition of 100 µM ATP increased the IK^+^ to values around 2.2 nA, while 4-aminopyridine (4-AP, 3 mM), a blocker of K_V_ channels, significantly reduced the ATP-induced IK^+^ from −20 mV ahead. Subsequent addition of iberiotoxin (IBTX, 100 nM), a specific blocker of high-conductance Ca^2+^-activated K^+^ (BK_Ca_) channels, almost abolished the IK^+^ (*n* = 5). (**B**) When tracheal myocytes were exposed to TES (40 nM, for 48 h), the ATP-induced IK^+^ were higher than those under control conditions. This effect was completely blocked by 4-AP, suggesting an important role of K_V_ channels in the TES potentiation effect. Further addition of IBTX (100 nM) almost annulled the remaining IK^+^ (*n* = 5). Symbols represent mean values ± S.E.M. A one-way analysis of variance followed by the Student–Newman–Keuls multiple comparison test was performed. In panel (**A**), * *p* < 0.05, ** *p* < 0.01 comparing ATP vs. control, 4-AP + ATP or 4-AP + IBTX + ATP groups; † *p* < 0.05 comparing control vs. 4-AP + ATP group; ¶ *p* < 0.05 comparing control vs. 4-AP + IBTX + ATP group. In panel (B), * *p* < 0.05, ** *p* < 0.01 comparing TES + ATP vs. TES, TES + 4-AP + ATP or TES + 4-AP + IBTX + ATP groups; ¶ *p* < 0.05, ¶¶ *p* < 0.01 comparing TES vs. TES + 4-AP + IBTX + ATP group.

**Figure 5:**
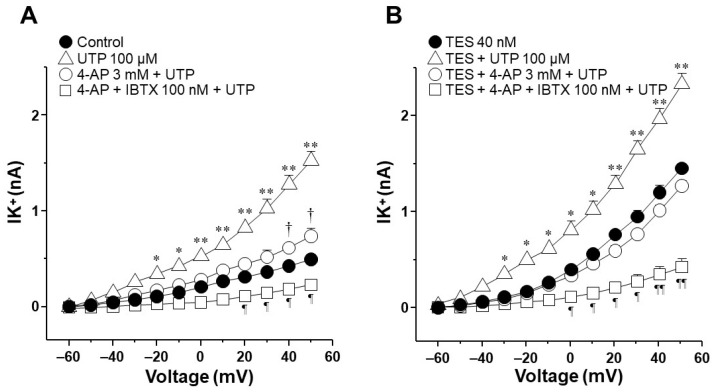
Testosterone (TES) enhancement of UTP-induced K^+^ currents involves delayed rectifier K^+^ (K_V_) channels. (**A**) In guinea pig tracheal myocytes, UTP 100 µM increased the outward K^+^currents (IK^+^) over the depolarizing pulses reaching about 1.6 nA (open triangle). UTP-evoked IK^+^ were markedly reduced by 4-aminopyridine (4-AP, 3 mM), a K_V_ channels blocker, from −20 mV onwards (*n* = 5). The remaining IK^+^ were abolished by the administration of iberiotoxin (IBTX, 100 nM), a specific blocker of high-conductance Ca^2+^-activated K^+^ (BK_Ca_) channels from +40 mV ahead. (**B**) Myocytes incubated with TES for 48 h showed an enhanced UTP-induced IK^+^ that were annulled with the perfusion of 4-AP 3 mM. Blockade of UTP-elicited IK^+^ with 4-AP demonstrates the involvement of K_V_ channels in the androgenic potentiation effect. Moreover, when IBTX 100 nM was added, the IK^+^ were almost attenuated (*n* = 5). The symbols represent the mean values ± S.E.M. After a one-way analysis of variance was performed, the Student–Newman–Keuls multiple comparison test was performed. In panel (**A**), * *p* < 0.05, ** *p* < 0.01 comparing UTP vs. control, 4-AP + UTP or 4-AP + IBTX + UTP groups; † *p* < 0.05 comparing control vs. 4-AP + UTP group; ¶ *p* < 0.05 comparing control vs. 4-AP + IBTX + UTP group. In figure (**B**), * *p* < 0.05, ** *p* < 0.01 comparing TES + UTP vs. TES, TES + 4-AP + UTP or TES + 4-AP + IBTX + UTP groups; ¶ *p* < 0.05, ¶¶ *p* < 0.01 comparing TES vs. TES + 4-AP + IBTX + UTP group.

To find out which type of K^+^ channel is involved in the TES effect, we used blockers of the K_V_ and the BK_Ca_ channels on the ATP- and UTP-induced K^+^ currents. In control tracheal smooth muscle cells, ATP and depolarizing pulses increased the K^+^ currents, which peaked at approximately 2.1 nA (Figure 4A, open triangle). The perfusion of 4-aminopyridine (4-AP, 3 mM, a blocker of K_V_ channels) partially decreased the ATP 100 µM-induced K^+^ currents from −20 mV ahead (open circle), but it did not reach the values of the control K^+^ currents caused by depolarizing pulses (closed circle). The summative addition of iberiotoxin (IBTX, 100 nM, the selective blocker of BK_Ca_ channels) decreased the remaining ATP-induced K^+^ currents from 30 mV forward (Figure 4A) below the control trace. These results confirm that the K^+^ current generated by ATP (along with the depolarizing pulses) is due to the opening of K_V_ and BK_Ca_ channels, as we previously demonstrated [27]. Moreover, as shown in Figure 2, TES 40 nM (for 48 h) significantly improved the ATP-induced K^+^ currents. 4-AP almost entirely suppressed the resulting K^+^ currents from −10 mV onwards, and IBTX blocked the remaining K^+^ currents (Figure 4B). The results were similar when the K^+^ currents were induced by UTP. Under control conditions, UTP 100 µM generated a K^+^ current that peaked at approximately 1.6 nA, and 4-AP partially reduced this K^+^ current from −20 mV ahead. At the same time, the consecutive addition of IBTX abolished the K^+^ currents elicited by UTP (Figure 5A). As shown in Figure 3, TES-treated myocytes showed a significantly augmented UTP-induced K^+^ current (Figure 5B), which was almost abolished by 4-AP from −30 mV forward. The consecutive addition of IBTX annulled the UTP-induced K^+^ currents. These results indicate that the effect of TES appears to be mediated mainly through the action of K_V_ channels.

### 2.4. In Tracheal Myocytes, K_V_ Currents but Not BK_Ca_ Currents Are Upregulated by Testosterone Treatment

**Figure 6:**
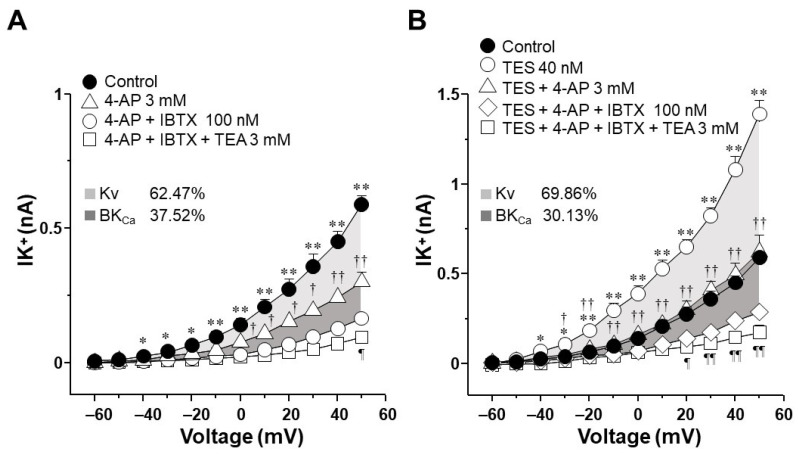
High-conductance Ca^2+^-activated K^+^ (BK_Ca_) channels and voltage-dependent delayed rectifier K^+^ (K_V_) channels are involved in the K^+^ currents induced by depolarizing pulses. In contrast, only K_V_ channels play a role when airway myocytes are incubated with testosterone (TES). (**A**) The protocol with depolarizing pulses from −60 to +50 mV in 10 mV increments produced outward K^+^ currents (IK^+^) that peaked at approximately 0.6 nA. 4-aminopyridine (4-AP, 3 mM), a K_V_ channels blocker, partially inhibited these currents. Consecutive addition of iberiotoxin (IBTX, 100 nM), a specific BK_Ca_ channels blocker, almost completely reduced the IK^+^. The remaining K^+^ currents were abolished by tetraethylammonium (TEA, 3 mM), a nonspecific blocker of K^+^ channels (*n* = 6). The area under the curve (AUC) illustrates in the light gray area that the K_V_ subtype accounts for 62.47% of the IK^+^ elicited by depolarizing steps, and BK_Ca_ for 37.52% (dark gray area). (**B**) Myocytes incubated with 40 nM TES (48 h) showed higher IK^+^ triggered by depolarizing pulses. The increase in IK^+^ by TES was prevented by 4-AP, and the addition of IBTX and TEA abolished the outward currents (*n* = 6). These results point out that TES is augmenting the expression of K_V_ channels. The AUC shows in the light gray area that the K_V_ subtype is responsible for 69.86% of the TES-induced enhancement of IK^+^, and BK_Ca_ for 30.13% (dark gray area). Symbols represent the mean ± S.E.M. For figures (**A**,**B**), a one-way analysis of variance followed by a Student–Newman–Keuls multiple comparison test was performed. In panel (**A**), * *p* < 0.05, ** *p* < 0.01 comparing control group vs. 4-AP, 4-AP + IBTX or 4-AP + IBTX + TEA groups; † *p* < 0.05, †† *p* < 0.01 comparing 4-AP vs. 4-AP + IBTX group; ¶ *p* < 0.05 comparing 4-AP + IBTX vs. 4-AP + IBTX + TEA group. In panel (**B**), * *p* < 0.05, ** *p* < 0.01 comparing TES group vs. control, TES + 4-AP, TES + 4-AP + IBTX or TES + 4-AP + IBTX + TEA groups; † *p* < 0.05, †† *p* < 0.01 comparing TES + 4-AP vs. TES + 4-AP + IBTX group; ¶ *p* < 0.05, ¶¶ *p* < 0.01 comparing TES + 4-AP + IBTX vs. TES + 4-A P+ IBTX + TEA group.

Because the K_V_ subtype seems to be more involved in the enhancement exerted by TES on ATP- and UTP-induced K^+^ currents compared to BK_Ca_ subtype, we decided to investigate the influence of this androgen on K^+^ currents generated only by the protocol of depolarizing pulses. In cultured tracheal myocytes without TES, depolarizing steps from −60 to +50 mV generated K^+^ currents that reached approximately 0.6 nA (Figure 6A). Administration of 4-AP blocked the K^+^ currents from −40 mV forward, and the subsequent addition of IBTX almost completely blocked the K^+^ currents from 0 mV forward (Figure 6A). Further administration of TEA annulled the K^+^ currents. These results indicate that although K_V_ and BK_Ca_ are the primary subtypes of K^+^ channels in guinea pig tracheal smooth muscle cells, K_V_ subtype is the major contributor to total K^+^ currents. Analysis of the area under the curve (AUC) showed that under the protocol of depolarizing steps, K_V_ was responsible for 62.47% and BK_Ca_ for 37.52% of the K^+^ currents mediated by these two subtypes of K^+^ channels. Additionally, the treatment of cultured myocytes with TES 40 nM increased the K^+^ currents generated by the depolarizing steps, as previously shown in Figure 2D and Figure 3D. Perfusion of 4-AP virtually abolished the enhancement of K^+^ currents generated by TES, and IBTX and TEA practically abolished the total K^+^ currents (Figure 6B). In this case, analysis of AUC showed that in myocytes treated with TES, the proportional current for K_V_ increased to 69.86%. In comparison, BK_Ca_ reduced its proportion to 30.13%. Therefore, the effect of the androgen relates exclusively to K_V_ channels by upregulating the function or the expression of these proteins.

### 2.5. Testosterone Increases the Expression of K_V_1.2 and K_V_1.5

**Figure 7:**
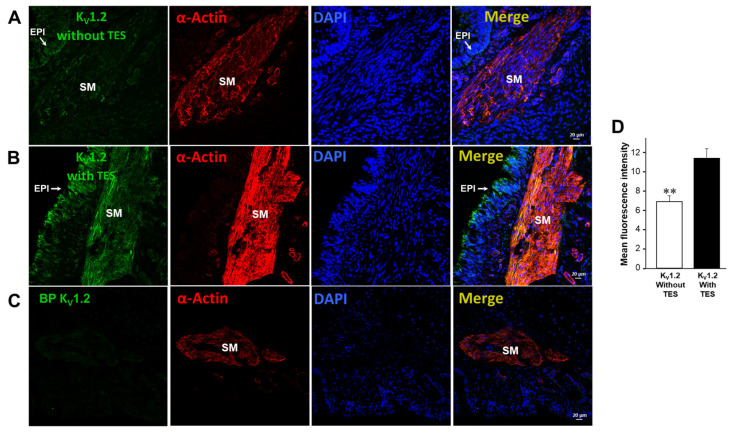
Testosterone increases the expression of K_V_1.2 in guinea pig airway smooth muscle. The first column of the figure displays K_V_1.2 immunofluorescence in guinea pig tracheal tissues without (**A**) and with (**B**) 40 nM TES (incubated for 48 h). The green color indicates the presence of K_V_1.2, and it can be noticed that exposure of tracheal tissues to TES augmented the fluorescence. In contrast, incubating the blocking peptide (BP) with anti-K_V_1.2 produced no fluorescence (**C**). The second column displays the smooth muscle α-actin in red, and the third column shows cell nuclei stained with DAPI in blue. In the fourth column, the images of the first three panels are merged. Additionally, the figure presents the quantification of K_V_1.2 expressed as mean fluorescence (**D**). The data show a significant increase in K_V_1.2 in the airway smooth muscle due to TES exposure. The bars in the graph represent the mean values ± the S.E.M. The significance was determined using an unpaired two-tailed *t*-test (** *p* < 0.01, *n* = 5). EPI stands for epithelium, and SM stands for smooth muscle. This figure was taken from our previous work [58].

**Figure 8:**
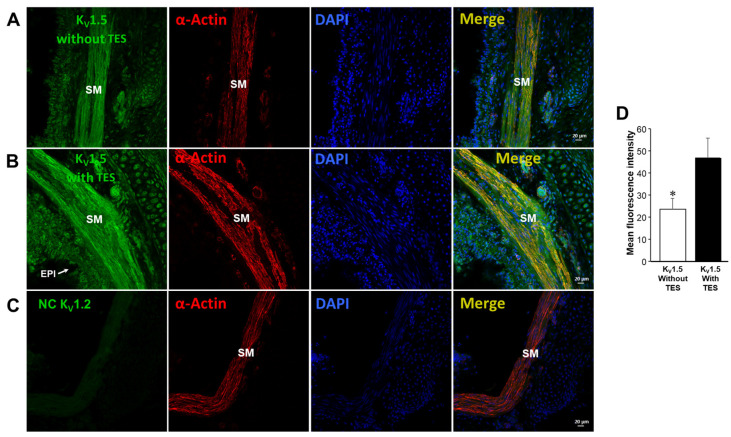
Testosterone induces the upregulation of K_V_1.5 in the airway smooth muscle of guinea pigs. The first column illustrates in green the immunofluorescence of K_V_1.5 in non-exposed guinea pig tracheal preparations (**A**) and tracheal tissues exposed to TES 40 nM for 48 h (**B**). TES-treated tracheal preparations showed increased fluorescence, whereas no signal was detected without a primary antibody for K_V_1.5 (NC = negative control) in (**C**). The second column shows smooth muscle α-actin in red, and the third column shows cell nuclei in blue. The first three panels are merged in the fourth column. The bar graph in (**D**) illustrates the quantification of K_V_1.5 expressed as mean fluorescence, *n* = 5. The bars in the graph represent the mean values ± the S.E.M. Significance was determined using an unpaired two-tailed *t*-test (* *p* < 0.05). The bar graph shows a significant increase in the levels of K_V_1.5 in response to TES. SM stands for muscle, and EPI stands for epithelium. This immunofluorescence was taken from our previous work [58].

Previous immunofluorescence assays confirmed that the expression of two subtypes of K_V_, K_V_1.2 and K_V_1.5, in the ASM of guinea pigs increased when tracheal rings were exposed to 40 nM TES for 48 h (Figure 7 and Figure 8) [58]. These results were analyzed by measurements of mean fluorescence intensity illustrated in Figure 7D and Figure 8D. Negative controls, including incubation with the corresponding blocking peptide or no primary antibody incubation, confirmed antibodies’ specificity against K_V_1.2 and K_V_1.5 proteins. No fluorescence signal was detected in the negative controls. The colocalization of the two K^+^ channels in the ASM was confirmed by detecting smooth muscle α-actin in Figure 7A,B and Figure 8A,B.

### 2.6. UTP Stimulates P2Y_2_ and P2Y_4_ Receptors in Airway Myocytes, and Testosterone Increases P2Y_4_-Mediated K^+^ Currents but Does Not Increase the Expression of P2Y_4_ Receptors

**Figure 9:**
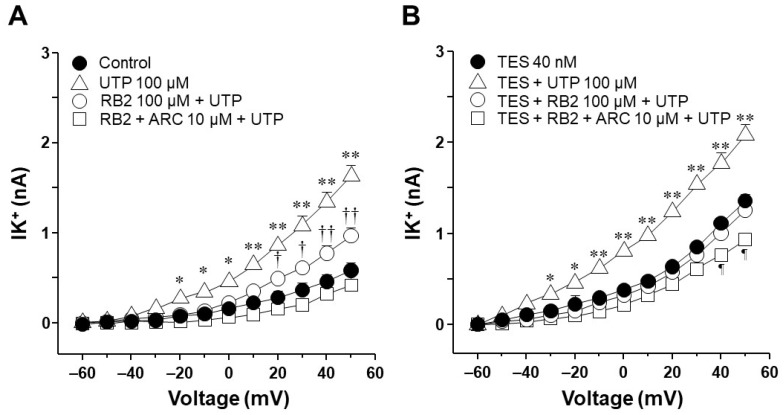
Role of P2Y_4_ receptors in the improvement of UTP-induced K^+^ currents caused by testosterone (TES). (**A**) Outward K^+^ currents (IK^+^) were elicited by depolarizing steps from −60 to +50 mV (control, closed circle). The IK^+^ increased in response to 100 µM UTP. This effect was partially attenuated by 100 µM RB2, which antagonizes the action of P2Y_1,4,6,11,12,13_ receptors except for P2Y_2_, and completely abolished by 10 µM AR-C118925XX, a specific P2Y_2_ receptor antagonist (*n* = 5). (**B**) In tracheal myocytes, UTP-induced IK^+^ increased significantly after 48 h of exposure to 40 nM TES (open triangle). These currents were blocked by 100 µM RB2, and the subsequent addition of 10 µM AR-C118925XX did not modify the IK^+^ (*n* = 5). These results suggest that the TES-mediated enhanced response to UTP involves P2Y_4_ receptors. Symbols represent mean values ± S.E.M. In figure (**A**), * *p* < 0.05, ** *p* < 0.01 comparing UTP vs. control, RB2 + UTP and RB2 + ARC + UTP groups; † *p* < 0.05, †† *p* < 0.01 comparing control vs. RB2 + UTP group. In figure (**B**), * *p* < 0.05, ** *p* < 0.01 comparing TES + UTP vs. TES, TES + RB2 + UTP and TES + RB2 + ARC + UTP groups; ¶ *p* < 0.05 comparing TES vs. TES + RB2 + ARC + UTP group. A one-way analysis of variance was performed, followed by a Student–Newman–Keuls test.

**Figure 10:**
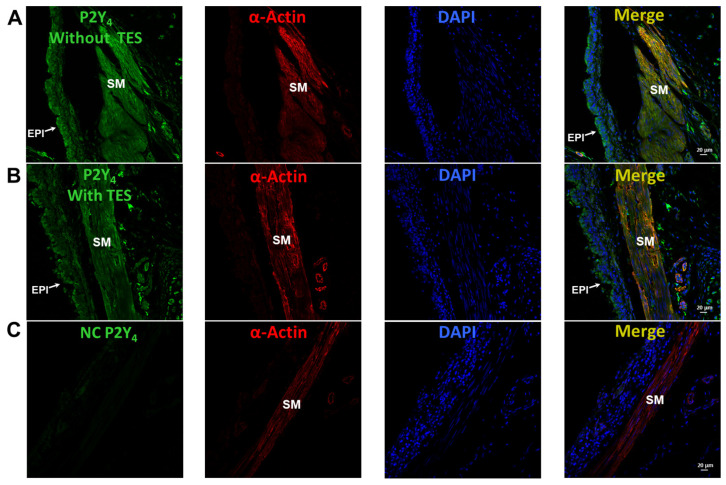
Testosterone (TES) does not modify the expression of P2Y_4_ receptors in airway smooth muscle. The first column shows preparations from guinea pig trachea, in which immunofluorescence for P2Y_4_ receptors in airway muscle is shown in green without (**A**) and with 40 nM TES incubated for 48 h (**B**). (**C**) The P2Y_4_ receptors did not fluoresce when no antibody was used (NC = negative control). The second column shows the α-actin of the smooth muscle (red), while the third column shows the cell nuclei (blue). Merged images from the first three panels are shown in the fourth column indicating the colocalization of P2Y_4_ receptors in the smooth muscle. SM = smooth muscle, EPI = epithelium.

In control tracheal myocytes, the antagonist for almost all P2Y receptors except P2Y_2_, RB2 100 μM [59,60,61,62], partially decreased the K^+^ currents induced by UTP (100 µM). Consecutive addition of AR-C118925XX (10 µM, selective antagonist of the P2Y_2_ receptors) [63] abolished the remaining UTP-induced K^+^ currents (Figure 9A). TES 40 nM enhanced the UTP-induced K^+^ currents, RB2 completely blocked this improvement, and AR-C118925XX did not modify these currents (Figure 9B). Based on these findings, we investigated whether TES could increase the expression of P2Y_4_ receptors in the ASM. However, immunofluorescence experiments revealed that TES did not improve the expression of P2Y_4_ receptors (Figure 10).

### 2.7. The Enhancement of ATP- and UTP-Mediated K^+^ Currents Depends on the G_S_ Protein–Adenylyl Cyclase Axis

**Figure 11:**
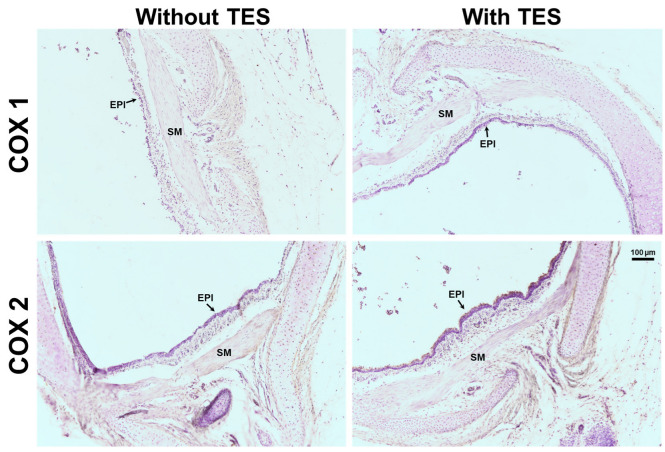
Chronic exposure to testosterone (TES) has no effect on cyclooxygenase (COX)-1 and COX-2 expression in guinea pig tracheal smooth muscle, as revealed by immunohistochemical staining. Tracheal rings exposed to 40 nM TES for 48 h showed no changes in the expression of COX-1 and COX-2 in airway smooth muscle (second column) compared to control tissues (first column). EPI = epithelium, SM = smooth muscle.

**Figure 12:**
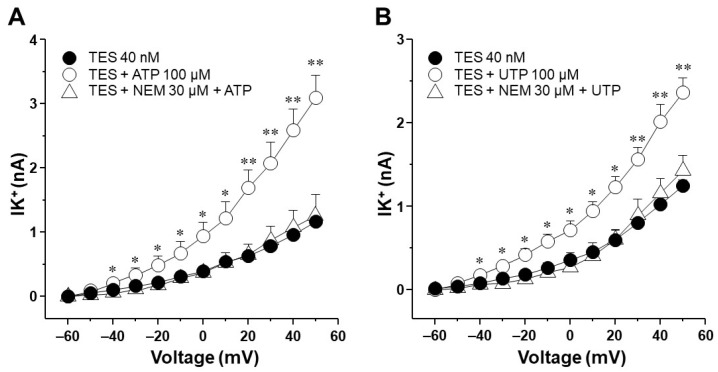
Importance of the activation of G proteins in the effect of testosterone (TES) on ATP- and UTP-induced K^+^ currents. Tracheal myocytes were subjected to step depolarizing pulses ranging from −60 to +50 mV in 10 mV increments. (**A**) ATP (100 µM) caused a significant increase in the outward K^+^ currents (IK^+^) in airway smooth muscle cells pre-treated with TES (40 nM). The addition of *N*-ethylmaleimide (NEM; 30 µM), which uncouples several receptors from G proteins, nullified this effect (*n* = 4). (**B**) Similarly, the myocytes pre-treated with TES showed an increase in the IK^+^ upon exposure to UTP (100 µM), which was also abolished by the addition of 30 µM NEM (*n* = 4). Symbols represent mean values ± S.E.M. In panel (**A**), * *p* < 0.05, ** *p* < 0.01 comparing TES vs. TES + ATP group. In panel (**B**), * *p* < 0.05, ** *p* < 0.01 when comparing TES vs. TES + UTP group. Repeated measure analyses of variance were performed, followed by Dunnett’s multiple comparison tests.

**Figure 13:**
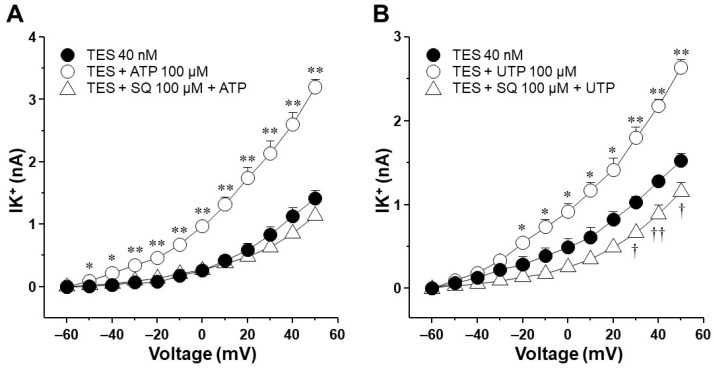
The improvement of K^+^ currents induced by ATP and UTP after incubation with testosterone (TES) requires adenylyl cyclase activity. Myocytes were subjected to step depolarization pulses ranging from −60 to +50 mV. (**A**) After 48 h of treatment with 40 nM TES, myocytes showed a significant increase in outward K^+^ currents (IK^+^) in response to 100 µM ATP. SQ 22,536 (SQ; 100 μM), an inhibitor of adenylyl cyclase, abolished this effect (*n* = 6). (**B**) The enhancement of the IK^+^ in myocytes pre-treated with TES 40 nM upon exposure to 100 µM UTP was blocked by the addition of 100 µM SQ, *n* = 7. Symbols represent mean ± S.E.M. In panel (**A**), * *p* < 0.05, ** *p* < 0.01 comparing the group of TES vs. TES + ATP group. In panel (**B**), * *p* < 0.05, ** *p* < 0.01 when comparing TES vs. TES + UTP group; † *p* < 0.05, †† *p* < 0.01 comparing the group of TES vs TES + SQ + UTP group. An analysis of variance followed by a Dunnett’s test was used for statistical analysis.

It has been postulated that the ATP- and UTP-induced K^+^ current increase and ASM relaxation are mediated by the production of prostaglandins in the smooth muscle, which in turn enhances cAMP signaling [27,36,37]. However, by performing immunostaining assays, we found that TES did not affect the expression of cyclooxygenase (COX)-1 or COX-2 (Figure 11), suggesting that prostaglandins are not involved in the action of the androgen. *N*-ethylmaleimide (NEM, 30 μM), a drug that uncouples G proteins from several receptors, blocked the increase in K^+^ currents elicited by ATP or UTP in tracheal myocytes previously incubated with TES, confirming that this effect occurs via receptors coupled to G proteins (Figure 12). Moreover, in another set of experiments, 100 μM SQ 22,536, a cell-permeable adenylyl cyclase inhibitor, completely abolished the enhanced ATP- and UTP-evoked K^+^ current increase (Figure 13). Overall, all these results point out that ATP and UTP in TES-treated myocytes stimulate P2Y_4_ receptors, which in turn activate a G protein, probably G_S_, and adenylyl cyclase to mediate the enhanced relaxation of the ASM.

## 3. Discussion

According to our findings, the chronic exposure of tracheal rings to 40 nM TES enhanced ATP- and UTP-induced relaxation involving the activation of K^+^ channels. TES increased ATP- and UTP-induced K^+^ currents in tracheal myocytes, and flutamide reversed this increase, confirming the genomic nature of these findings. Moreover, the effect of TES on ATP- and UTP-induced K^+^ currents was abolished by 4-AP, indicating that K_V_ channels are involved in the androgenic potentiation effect. The upregulation of K_V_ currents elicited by depolarizing pulses in myocytes chronically exposed to TES is explained by the increased expression of K_V_1.2 and K_V_1.5 induced by the androgen. RB2, the antagonist of almost all P2Y receptors (except for P2Y_2_), inhibited the UTP-induced increase in K^+^ currents, suggesting that P2Y_4_ receptors are implicated in the action of the androgen. However, TES did not affect P2Y_4_ receptor expression or COX-1 and COX-2 in the ASM. These last findings ruled out the involvement of prostaglandins. The stimulation of P2Y_4_ receptors with ATP or UTP triggers the G protein–adenylyl cyclase pathway, which TES could positively regulate. Consequently, the probability of K^+^ channels opening increases, leading to membrane hyperpolarization and smooth muscle relaxation.

ATP relaxes the ASM of several species, including mice, rats, rabbits, guinea pigs, and humans [31,36,37,64]. Moreover, we have demonstrated that ATP-induced ASM relaxation is dependent on P2Y_2_ and P2Y_4_ receptors signaling [27]. In the present study, we confirmed that ATP and UTP relaxed the histamine-pre-contracted ASM of a guinea pig in a concentration-dependent manner (Figure 1). We also found that a physiological concentration of TES (40 nM) significantly increased the relaxation response for both agonists (Figure 1). Furthermore, we observed that ATP caused a greater relaxation of tracheal tissues compared to UTP in both the control and TES groups. The difference in the relaxation of the ASM triggered by ATP or UTP may be due to the involvement of the hydrolysis of ATP and the activation of other purinergic receptors [27]. For example, adenosine, produced by the degradation of ATP by ectonucleotidases, activates A_2_ receptors, a subgroup of the purinergic P1 receptor family. This process facilitates the relaxation of the ASM pre-contracted with KCl or histamine [65,66]. Our previous work has shown that blocking ectonucleotidases in guinea pig tracheal rings with ARL 67156 shifted the concentration–response curve to ATP to the left; i.e., the relaxation response was more intense [27]. This shift indicates that ATP is enzymatically degraded under our experimental conditions. Moreover, we also showed a remanent relaxation induced by ATP even when P2Y_2_ and P2Y_4_ receptors were blocked [27]. These previous results suggest that the higher relaxation induced by ATP than UTP may be mediated by adenosine acting through P1 receptors [67].

Several studies have convincingly demonstrated the direct influence of TES on the relaxation of ASM. There are different mechanisms through which this hormone can affect ASM tension, including the inhibition of membrane Ca^2+^ channels [45]. For example, androgens induce the relaxation of pre-contracted guinea pigs and bovine tracheal smooth muscle by blocking L-type voltage-dependent Ca^2+^ channels (L-VDCCs) and store-operated Ca^2+^ channels (SOCCs) [51,52]. In addition, androgenic hormones enhance the relaxation of ASM caused by β adrenergic agonists [49,50], and TES induces ASM relaxation via an epithelial-dependent mechanism that is related to nitric oxide production [48]. All these reported effects are mediated through non-genomic pathways triggered by the androgens. On the other hand, we recently demonstrated the genomic effects of TES on ASM relaxation. This androgen significantly augmented the relaxation caused by the clinically used bronchodilators, salbutamol and theophylline, in guinea pig tracheal rings by upregulating the β_2_-AR and K^+^ channels [40,58,68]. In the experiments shown in the present work, a 48 h incubation of the androgen was performed, suggesting that the enhanced relaxation evoked by ATP and UTP may be due to a genomic pathway. Moreover, because K^+^ channels are implicated in the purinergic-mediated guinea pig ASM relaxation [27], we investigated whether these channels are involved in the increased ATP- and UTP-induced relaxation. Thus, we blocked the K^+^ channels with TEA, which reversed the effect of the androgen (Figure 1).

Next, we explored the influence of TES on K^+^ currents triggered by ATP and UTP. We found that myocytes chronically exposed to TES (40 nM) showed increased K^+^ currents elicited by depolarizing pulses (Figure 2D and Figure 3D) and by increasing concentrations of ATP (Figure 2E–H) and UTP (Figure 3E–H). The fact that TES markedly augmented the K^+^ currents during depolarizing pulses alone indicates the direct regulation of K^+^ channels by the androgen. Moreover, the enhanced ATP- and UTP-evoked K^+^ currents suggest a modulation of the purinergic signaling. To confirm the possible genomic mechanism of TES, we used flutamide, an androgen receptor antagonist, which abolished the sex-hormone-induced effects on the K^+^ currents (Figure 2 and Figure 3). Thus, it is conceivable that TES upregulates the expression of proteins involved in purinergic responses, including P2Y receptors and K^+^ channels. The major populations of K^+^ channels involved in the modulation of the tone and relaxation of the ASM are the K_V_ and BK_Ca_ [27,40,56,58,69]. Therefore, we used 4-AP and IBTX to investigate which type of K^+^ channel is responsible for the TES effect on ATP- and UTP-induced K^+^ currents. As shown in Figure 4A and Figure 5A, ATP- and UTP-induced K^+^ currents in control myocytes (without TES) were partially decreased by 4-AP, and the remaining K^+^ currents were abolished by the addition of IBTX. Consistent with the results in Figure 2 and Figure 3, the K^+^ currents were greater when cells were incubated with TES (Figure 4B and Figure 5B). Notably, 4-AP completely blocked the TES-induced enhancement of K^+^ currents, highlighting the importance of K_V_ channels in this phenomenon. Given these findings, we decided to investigate the effects of this androgen on the K^+^ currents produced only by the protocol of depolarizing steps. First, we demonstrated that K_V_ and BK_Ca_ channels were involved in the K^+^ currents by 62.47% and 37.52%, respectively, under control conditions (Figure 6A). However, exposure to TES increased the contribution of K_V_ channels (69.86%) but decreased that of BK_Ca_ channels (30.13%) (Figure 6B). These last results of Figure 6 confirm the positive regulation of K_V_ channels by the androgen. In this context, we had previously shown that TES increased the expression of K_V_1.2 and K_V_1.5 isoforms, but not BK_Ca_, in the ASM (Figure 7B and Figure 8B) [58]. In this context, there is evidence of the genomic effects of TES on K^+^ channels in cardiomyocytes, where this hormone improved the expression of K_V_7.1 [70]. On the other hand, there are no reports of non-genomic effects of TES on K^+^ channels in ASM. However, in vascular smooth muscle, the acute application of TES resulted in vasodilation associated with opening K_V_ and BK_Ca_ channels [71,72]. Further research is needed to determine whether TES can trigger non-genomic mechanisms affecting K^+^ channels in the ASM.

Additionally, we investigated the possible effects of TES on P2Y_2_ and P2Y_4_ receptors, as ATP exerts its relaxing effect via their activation [27]. In patch-clamp studies, the K^+^ currents elicited by UTP were prevented in control cells by pharmacologically blocking P2Y_4_ and P2Y_2_ receptors (Figure 9A). We used RB2 as an antagonist of P2Y_4_ receptors because this chemical compound can block the action of almost all P2Y receptors, including P2Y_4_, except for P2Y_2_ [59,61]. AR-C118925XX was employed as a selective antagonist of P2Y_2_ [63]. We focused on UTP as an agonist of P2Y receptors to avoid the nonspecific effects of ATP on other purinergic receptors [61,62,73,74,75]. Long-term TES exposure significantly increased the UTP-evoked K^+^ currents, and RB2 antagonized this effect (Figure 9B). Therefore, we investigated the possibility that TES might increase the expression of this receptor in the ASM, but no changes in the immunofluorescence were observed (Figure 10). It is most likely that the increase in ATP- and UTP-induced K^+^ currents is exclusively due to the upregulation of K^+^ channels, specifically K_V_1.2 and K_V_1.5.

Previous evidence postulates that ATP-induced relaxation of ASM is caused by intermediate biosynthesis of PGE_2_ following stimulation of P2Y_2_ and P2Y_4_ receptors [27,36]. The activation of these receptors triggers heterotrimeric G_q_ proteins [30,76,77] leading to intracellular Ca^2+^ increases [14,78]. Ca^2+^ mobilization could activate phospholipase A_2_ (PLA_2_), which favors PGE_2_ production [79]. Because COX is the main pathway that catalyzes the conversion of arachidonic acid to prostaglandins, we investigated whether TES could enhance the expression of this enzyme. Chronic exposure to TES in tracheal slices did not increase the expression of constitutive COX-1 and inducible COX-2 in ASM (Figure 11), indicating that the androgen effect is not related to PGE_2_ synthesis.

We then decided to examine the signaling pathway that modulates the increase in K^+^ currents triggered by ATP and UTP in myocytes exposed to TES. Perfusion of NEM (a G protein uncoupler) and SQ 22,536 (an adenylyl cyclase inhibitor) reversed the effect of TES on the K^+^ currents generated with ATP and UTP (Figure 12 and Figure 13). In general, P2Y_4_ receptors trigger the G_q_-mediated signaling pathway with the subsequent activation of phospholipase C_β_ and the intracellular Ca^2+^ increase [14,80]. Nevertheless, it has been reported that several P2Y receptors can couple to different G proteins [80]. For instance, the P2Y_11_ receptor that becomes active upon ATP binding forms connections with members of the G_q_ and G_s_ subfamilies [80,81,82]. G_s_ proteins initiate the activation of adenylyl cyclase, leading to augmented cAMP levels. The interaction between the P2Y_11_ receptor and G_s_ proteins holds significance in regulating various physiological processes, including cell migration and apoptosis [83,84,85]. In this context, we hypothesize that the P2Y_4_ receptor in the ASM couples to a G_s_ protein after TES exposure which would lead to the formation of cAMP. The synthesis of cAMP catalyzed by AC promotes the activation of PKA and the phosphorylation of the K_V_, increasing its activity [41,86,87]. This might explain why NEM and SQ 22,536 abated the effect of TES. Nonetheless, this hypothesis requires further corroboration. In addition, it has been demonstrated that P2Y_4_ can couple to the βγ-subunits of G_i/o_ proteins in rat sympathetic neurons [77]. G_i_ activation classically leads to reduced cAMP synthesis and inhibition of G_s_-stimulated AC activity [88,89,90]. However, it has been suggested that the βγ-subunits of the G_i_ protein can indirectly trigger the activity of the AC (type VI) in HEK cells [91]. This AC isoform is the predominant one in ASM, but we previously showed that the expression of this enzyme was not altered in guinea pig tracheal tissues exposed to TES [40]. Collectively, our results indicate that TES enhances the P2Y_4_ signaling pathway (probably via a G_S_ protein) and increases the expression of K_V_ channels. Activation of this purinergic receptor induces the formation of cAMP and the opening of K_V_ channels, which are enhanced by TES, resulting in augmented ASM relaxation.

In the context of airway inflammation in asthma patients, ATP is significantly released into the airways [13]. In this regard, platelets are an important source of ATP during the asthmatic process, as they can store up to 1 M in their granules [92]. There is evidence that allergen exposure triggers intravascular platelet activation in patients with allergic asthma [93] and in the pulmonary capillaries of ovalbumin-sensitized mice [94], emphasizing the significant role of circulating activated platelets in the progression of allergic airway inflammation following exposure to allergens. Our studies with guinea pig platelets have confirmed this phenomenon by demonstrating the release of ATP during antigen challenge [3]. In addition to platelets, other entities, such as airway epithelial cells [21] and ASM cells [28], are also involved in ATP release during inflammation. Extracellular ATP exerts direct effects on the ASM by inducing contraction and facilitating the recruitment of inflammatory cells into the airways [17,28,95,96]. These cells can subsequently release bronchoconstrictor substances such as histamine, thromboxane, leukotrienes, and acetylcholine [97,98,99,100,101]. Given the pre-existing smooth muscle contraction during asthma, ATP probably promotes relaxation. Thus, ATP may serve as a regulating factor that prevents excessive bronchoconstriction and maintains airway patency during an asthma attack, particularly in young men when TES levels are elevated. Furthermore, studies in animal and human tissues have shown that K^+^ channel openers can induce hyperpolarization of ASM cells, resulting in bronchodilation and attenuation of airway hyperresponsiveness [57,102,103,104,105,106,107,108]. Therefore, K_V_ channels might be a promising pharmacologic target for a novel and effective therapeutic approach in the treatment of asthma in young men.

## 4. Materials and Methods

### 4.1. Experimental Animals

Male Hartley guinea pigs weighing between 350 and 400 g and aged 4–6 weeks were used. The animals were reared under standard institutional conditions, i.e., filtered air, 21 ± 1 °C, 50–70% humidity, sterile bedding, and feed consisting of Harlan^®^ pellets and sterilized water. The General Health Law for Health Research (NOM-062-Z00-1999) and the Mexican National Animal Welfare Laws establish guidelines that were followed when handling the animals. The scientific and bioethical committees of the National Autonomous University of Mexico approved the project under the number FM/DI/003/2020.

### 4.2. Organ Baths

Pentobarbital sodium (50 mg/kg) was used to anesthetize the guinea pigs before exsanguination. The tracheas were dissected and placed in a dissection chamber filled with Krebs-Ringer solution containing [mM] 118 NaCl, 25 NaHCO_3_, 11 glucose, 4.6 KCl, 2 CaCl_2_, 1.2 KH_2_PO_4,_ and 1.2 MgSO_4_. The solution was bubbled with a carbogen mixture of 95% O_2_ and 5% CO_2_ for 30 min, keeping the pH constant in the physiological range (7.3 to 7.4). Afterward, the fatty and connective tissue was removed from the trachea. The tracheas were divided into seven segments, each with four cartilage rings. The tissues were incubated without and with TES (40 nM) for 48 h at 9 °C in separate Eppendorf tubes containing 1 mL of Krebs-Ringer solution bubbled for 1 h with 5% CO_2_ in oxygen. After 48 h of incubation, each tissue was hung on a transducer at a resting tension of 1 g in separate organ baths containing 5 mL Krebs-Ringer solution at 37 °C and continuously bubbled with 5% CO_2_ in oxygen. A CyberAmp 380 signal integrator system (Axon Instruments, Foster City, CA, USA) and a Digidata 1440A interface (Axon Instruments) were used to record isometric force with a model FT03 strain transducer (Grass Instruments, West Warwick, RI, USA). Guinea pig tracheal smooth muscle contraction was recorded, and data were acquired on a computer using Axoscope software version 10.2 (Axon Instruments, Foster City, CA, USA). The rings were in resting tension for 1 h before the experimental procedures were performed. The contractile apparatus was optimized, and tissue responses were normalized by stimulating the rings with KCl (60 mM). The preparations with or without TES were pre-contracted with histamine (10 µM). Once maximal contraction was achieved, a cumulative concentration–response curve was generated with ATP or UTP (1, 3.2, 10, 32, 100, 320, and 1000 µM) to elicit and study ASM relaxation. In one experimental group, the tracheal rings were incubated with TEA (1 mM), 15 min before the addition of ATP or UTP.

### 4.3. Patch-Clamp Studies

The guinea pig trachea was removed as described in the procedure for organ baths. The connective tissue and epithelium were removed from the tracheal smooth muscle and subjected to enzymatic disruption by placing it in 5 mL of Hanks solution (GIBCO, Waltham, MA, USA) containing 0.05 U/mL papain (Worthington Biochemical Corporation, Lakewood, NJ, USA) and 2 mg L-cysteine. The pH of the solution was adjusted to 7.4 with 1 M NaHCO_3_, and the tissues were incubated at 37 °C for 10 min. The muscle strip was then washed with Leibovitz solution (L15, GIBCO) to remove excess enzymes and then placed in Hanks solution containing 1 mg/mL collagenase type I (Worthington Biochemical Corporation) and 1 mg/mL collagenase type II (Worthington Biochemical Corporation) for 10 min at 37 °C. The tissue was then aspirated approximately 25 times with a Pasteur pipette to allow the dissociation of the myocytes. The enzymatic activity was stopped with the L15 solution, and the cells were centrifuged at 600 rpm for 6 min. The supernatant was removed, and the last step was repeated once. The cell pellet was re-suspended in 6 mL minimal essential medium (MEM, GIBCO) containing 5% fetal bovine serum, 10 mM glucose, 2 mM L-glutamine, 10 µg/mL streptomycin, and 10 U/mL penicillin. Myocytes were cultured on coverslips impregnated with sterile rat tail collagen placed in 6-well plates. A group of myocytes was incubated with 40 nM TES (48 h) or 3.2 µM flutamide 30 min prior to the addition of TES. The cells were stored in a 5% CO_2_ incubator at 37 °C for 48 h before patch-clamp experiments were performed.

The coverslip containing the cultured tracheal myocytes was placed in a chamber that was continuously perfused with a volume of 0.5–1 mL of external solution at a flow rate of 1.5–2 mL/min. For K^+^ currents measurement, the external solution was adjusted to pH = 7.4 with NaOH and had the following composition (mM): 130 NaCl; 10 HEPES; 10 glucose; 5 KCl; 3 NaHCO_3_; 1.2 KH_2_PO_4_; 1 CaCl_2_; 0.5 MgCl_2_; 0.1 niflumic acid. The perfusion chamber was adapted to an inverted microscope (Zeiss model IP03). The patch pipettes for voltage recording were made of borosilicate glass (1B200F-6, World Precision Instruments, Sarasota, FL, USA) using a micropipette puller (P-87, Sutter Instruments Co., Novato, CA, USA). The pipette resistance was between 3 and 6 MΩ. K^+^ currents measurements were recorded with an Axopatch amplifier (model 200A, Axon Instruments) and performed with the 1–5 KHz current filters at a frequency of 10 KHz. The composition of the patch pipette internal solution was (mM) 140 K^+^ gluconate, 5 NaCl, 5 HEPES, 5 ATP, 1 EGTA, 0.1 leupeptin, and 0.1 GTP, adjusted with KOH to pH = 7.3.

Outward K^+^ currents were recorded using pClamp v10.2 software (Axon Instruments) with a protocol consisting of depolarizing pulses from −60 to +50 mV in steps of 10 mV with a holding potential of −60 mV for 500 ms at 1 Hz. Myocytes were perfused with cumulative concentrations of ATP or UTP (1, 10, 100, and 1000 µM) and received the protocol of depolarizing pulses. To characterize K^+^ channels in a group of experiments, cells were perfused with 3 mM 4-AP and 100 nM IBTX, which block K_V_ and BK_Ca_, respectively. TEA was used to block K^+^ channels nonspecifically. Some airway myocytes were perfused with Reactive Blue 2 (RB2, 100 μM) and AR-C118925XX (10 µM) before the addition of 100 µM UTP. In a series of experiments, 30 μM *N*-ethylmaleimide (NEM; a compound that uncouples G proteins) and SQ 22,536 (100 μM, adenylyl cyclase inhibitor) were perfused for 10 min before the addition of 100 µM ATP or UTP.

### 4.4. Double Immunofluorescence

Tracheal tissues were fixed in 4% paraformaldehyde in phosphate-buffered saline (PBS) for 4 h after gradual dehydration in 30% sucrose/PBS for 17 h before being included in O.C.T. compound (Tissue Tek, Torrance, CA, USA) and frozen at 60 °C. Frozen sections (10 nm thick) were obtained with a cryostat (Ecoshel ECO-1900, Pharr, TX, USA) and mounted on gelatinized slides. Afterward, slides were transferred into a Sequenza slide rack (Thermo Fisher Scientific, Rockford, IL, USA), washed with TBS, and permeabilized with 0.001% Tween 20 in TBS. To block nonspecific binding to proteins, cryosections were treated with 10% horse serum for 2 h at room temperature. Then, the sections were incubated with the primary antibody anti-P2Y_4_ diluted 1:75 overnight at 4 °C (Alomone Labs, APR-006, Jerusalem, Israel). The secondary antibody Alexa488 donkey anti-rabbit IgG (Life Technologies, Foster City, CA, USA) was incubated for 20 min (1:500). Anti-α-actin (1A4) Alexa Fluor 546 (sc-32251 AF546, Santa Cruz Biotechnology, Dallas, TX, USA), diluted 1:1200, was incubated overnight at 4 °C as the next primary antibody. The nuclei in all tissue slices were counterstained with 4′, 6-diamidino-2-phenylindole (DAPI, Life Technologies). Slides were maintained with Fluoromount aqueous mounting medium (Merck, Darmstadt, Germany). Immunofluorescence was observed using an LSM 880 Zeiss confocal microscope (Oberkochen, Germany). For display purposes, images in which P2Y_4_ is green, actin is red, and nuclei are blue were merged.

### 4.5. Immunochemistry

Tracheas from guinea pigs were obtained as described above and then incubated with and without TES 40 nM for 48 h at 9 °C. Then, tracheal tissue was fixed in 4% paraformaldehyde for 4 h and placed in 30% ethanol until their inclusion in paraplast plus (Sigma Aldrich Co., St. Louis, MO, USA). Once paraffinized, tissue blocks were cut into thin slices (5 μm) and placed over gelatinized slides. Paraffin was removed by incubation at 60 °C and placed in xylol, followed by graded alcohols, and then slices were washed with Tris-buffered saline (TBS). Slices were transferred into a Sequenza slide rack, washed with TBS, and permeabilized with 0.5% Triton/TBS. To block nonspecific binding to proteins, 10% horse serum was applied to the slices for 2 h at 32 °C. The sections were incubated with Bloxall blocking solution (Vector Laboratories, Burlingame, CA, USA) for 20 min at room temperature. The slides were incubated overnight at 4 °C with the rabbit polyclonal antibody to COX-1 (sc-7950, Santa Cruz Biotechnology, CA, USA) or COX-2 (sc-7951,) both diluted 1:25. For negative control, the primary antibody was omitted. After washing with TBS, the sections were incubated with donkey anti-rabbit IgG HRP (Thermo Fisher Scientific, Rockford, IL, USA) for 20 min and washed with TBS. Finally, the Betazoid DAB Chromogen Kit (Biocare Medical Pike Lane, CA, USA) was added to each section for 4 min. All slices were counterstained with hematoxylin (Biocare Medical Pike Lane, CA, USA) and maintained with a Sub-X mounting medium (Electron Microscopy Sciences, Hatfield, PA, USA). Images were collected and processed with a Nikon eclipse Ni microscope (Tokyo, Japan). For display purposes, images in which COX-1 or COX-2 is brown and nuclei are blue were merged.

### 4.6. Drugs and Chemicals

Testosterone (17β-hydroxy-4-androsten-3-one, TES), flutamide, histamine, ATP, UTP, TEA, RB2, *N*-ethylmaleimide, L-cysteine, and SQ 22,536 were purchased from Sigma Chem. Co. (St. Louis, MO, USA). 4-aminopyridine was purchased from Research Chemical Ltd. (Word Hill, MA, USA), iberiotoxin from Enzo Life Sciences (Farmingdale, NY, USA), and AR-C118925XX from Tocris Bioscience (Avonbridge Trading Estate, Bristol, UK). TES, flutamide, and 4-aminopyridine were diluted in absolute ethanol, and the highest percentage used was 0.1% *v*/*v* of vehicle. AR-C118925XX was dissolved in dimethyl sulfoxide (DMSO) 0.1% *v*/*v*.

### 4.7. Statistical Analysis

In organ baths, Dunnett’s multiple comparison test was performed after a one-way analysis of variance to examine these data. K^+^ currents were analyzed using a one-way analysis of variance, followed by Student–Newman–Keuls test or Dunnett’s test. In the experiments, each value of “n” refers to a different animal. Data are presented as mean ± the standard error of the mean (S.E.M). Statistical significance was set at *p* < 0.05.

## 5. Conclusions

In summary, our studies demonstrate that chronic exposure of guinea pig ASM to a physiological concentration of TES leads to a ~50% increase in ATP- and UTP-elicited K^+^ currents. This increase is associated with the enhanced signaling of P2Y_4_ receptors and augmented expression of K_V_ channels. These mechanisms result in the improvement of ATP- and UTP-induced ASM relaxation by 22.17% and 12.31%, respectively. The enhanced ASM relaxation may limit the severity of bronchospasm in teenage boys and men. To test this hypothesis, further experiments using plethysmography to measure airway resistance and evaluate bronchorelaxation induced by ATP are needed. However, these experiments are beyond the scope of the present work. Additionally, the activation of G_S_ proteins after P2Y_4_ receptor stimulation suggested in this article should be further corroborated.

## Data Availability

Data are available upon request.
